# Statin Co-Administration Is Associated with Subclinical Echocardiographic Alterations and Relative Change Amplitude of Estimated Systolic Pulmonary Artery Pressure After Anthracycline Chemotherapy: A Retrospective Cohort Study

**DOI:** 10.3390/jcm15187105

**Published:** 2026-09-13

**Authors:** Shouzhi Wang, Ying Yu, Lei Cao, Jiawei Xiong, Chuang Yin, Nianan He

**Affiliations:** 1 Department of Ultrasound, The First Affiliated Hospital of USTC, Division of Life Sciences and Medicine, University of Science and Technology of China, Hefei 230001, China; 2 Department of Pharmacy, The First People’s Hospital of Hefei, The Third Affiliated Hospital of Anhui Medical University, Hefei 230061, China

**Keywords:** anthracycline-induced cardiotoxicity, statins, estimated systolic pulmonary artery pressure change, left ventricular function, cardio-oncology

## Abstract

**Background:** Anthracycline-induced cardiotoxicity remains a major clinical challenge in oncology. The association between statin use and pulmonary hemodynamic changes during anthracycline chemotherapy remains largely unexplored. **Methods:** The primary endpoint was change in left ventricular ejection fraction from baseline to 12 months. Secondary endpoints included changes in left ventricular end-systolic volume index and mean e’ velocity. Exploratory endpoints included changes in other diastolic parameters, all estimated systolic pulmonary artery pressure (SPAP)-derived variables, and the descriptive incidence of cancer therapeutics-related cardiac dysfunction (CTRCD). Analyses included multivariable linear regression, 1:1 propensity score matching with baseline echocardiographic parameters included in the matching model, and multiple imputation for missing SPAP data. **Results:** After confounder adjustment, statin use was independently associated with a milder decline in mean e’ velocity (*β* = 0.75 cm/s, 95% CI 0.41 to 1.09, *p* < 0.001) and a smaller reduction in E/A ratio (*β* = 0.06, 95% CI 0.01 to 0.11, *p* = 0.02). No significant between-group difference was observed for adjusted ejection fraction change (*β* = 0.28%, 95% CI −1.11 to 1.66, *p* = 0.694). Statin use was associated with lower relative change amplitude of estimated SPAP (*β* = −4.55%, 95% CI −7.34 to −1.76, *p* = 0.001), with no significant effect on signed mean change. A descriptive component analysis showed a smaller signed mean change in estimated right atrial pressure in the statin group, with no significant difference in tricuspid regurgitant pressure gradient change. This comparison cannot mathematically decompose the absolute-change SPAP endpoint. The incidence of study-defined CTRCD was numerically lower in the statin group (1.25% vs. 4.8%, OR 0.25, 95% CI 0.05 to 1.28, *p* = 0.15), but this difference was not statistically significant and the study was underpowered for this clinical endpoint. **Conclusions:** In this retrospective cohort, concomitant statin therapy was associated with smaller changes in diastolic parameters and lower relative change amplitude of estimated SPAP between baseline and 12 month follow-up. No significant independent association with left ventricular systolic function was observed after multivariable adjustment. These hypothesis-generating findings require confirmation in appropriately powered prospective multicenter studies before any clinical implications can be considered.

## 1. Introduction

Anthracyclines are widely used to treat diverse malignancies. Their use is associated with a well-documented risk of dose-dependent cardiotoxicity. This toxicity can manifest acutely during treatment or as late-onset cardiomyopathy years later [1]. The central mechanisms involve topoisomerase IIβ-mediated DNA double-strand breaks, mitochondrial dysfunction, and excessive reactive oxygen species generation [2]. Dexrazoxane is currently the only FDA-approved cardioprotective agent for reducing the risk of anthracycline-associated cardiomyopathy. Its use is generally considered in patients receiving higher cumulative doxorubicin doses, often above 300 mg/m^2^ [3], though clinical decisions also incorporate individual patient risk factors, treatment regimen, and expected oncologic benefit. Emerging safety concerns, including reports of coagulation disorders, have also limited its broader use in routine practice [4]. A broader patient population therefore requires strategies for mitigating cardiotoxicity risk, highlighting the need for accessible, well-tolerated agents that can be integrated into routine oncological care without restrictive eligibility criteria [5].

Previous studies on anthracycline-induced cardiotoxicity have predominantly focused on the left ventricle. Systolic pulmonary artery pressure (SPAP), a key indicator of pulmonary circulation, can be estimated noninvasively via tricuspid regurgitation peak velocity combined with the simplified Bernoulli equation [6]. Elevated SPAP increases right ventricular afterload and may promote biventricular dysfunction through ventricular interdependence mechanisms, including interventricular septal shift and shared pericardial constraint. However, SPAP itself reflects right ventricular pressure load rather than direct right ventricular systolic performance [7]. Direct assessment of right ventricular function provides comprehensive information, but SPAP monitoring serves as a readily available surrogate that adds clinically useful insights to standard left ventricular evaluation.

Statins have been investigated as agents potentially associated with favorable cardiac changes during chemotherapy. Their effects may extend beyond lipid lowering and involve multiple cardiovascular pathways [8,9]. At the myocardial level, preclinical studies suggest that statins may reduce cardiomyocyte apoptosis, oxidative stress, and fibrosis through modulation of several signaling cascades. At the vascular level, experimental data indicate that statins may improve endothelial function and modulate pulmonary vasomotor tone through inhibition of the RhoA/Rho-kinase pathway [10,11,12,13,14]. These pleiotropic effects could theoretically buffer chemotherapy-induced endothelial injury and reduce the magnitude of hemodynamic change without necessarily altering mean pressure levels. None of these pathways were directly measured in the present clinical cohort.

Several meta-analyses have reported an association between statin therapy and lower risk of anthracycline-related cardiac dysfunction [8,9]. At the randomized controlled trial level, the STOP-CA trial reported that atorvastatin was associated with lower incidence of anthracycline-related cardiac dysfunction in lymphoma patients [15]. In contrast, the SPARE-HF and PREVENT trials did not demonstrate an association between statin use and early post-chemotherapy left ventricular ejection fraction (LVEF) decline [16,17]. Real-world propensity-score-matched studies further support an association between statin exposure and lower heart failure risk [18].

The current evidence on statin-associated cardiac changes derives almost exclusively from left ventricle focused studies. The potential association between statin use and pulmonary hemodynamic changes during anthracycline chemotherapy remains insufficiently characterized. None of the molecular pathways described above were directly measured in clinical cohorts, and all mechanistic inferences therefore remain hypothetical.

This study evaluates the association between concomitant statin therapy and echocardiographic changes in patients receiving anthracycline-based chemotherapy. The primary aim is to compare left ventricular functional changes between statin users and non-statin users. A secondary aim is to examine whether statin co-therapy is associated with smaller changes in pulmonary hemodynamic parameters. The potential co-variation between pulmonary pressure changes and left ventricular function is explored as a hypothesis-generating observation. The findings may offer new insights into statin-associated echocardiographic changes and inform more comprehensive cardiac surveillance strategies for patients receiving anthracycline-based chemotherapy.

## 2. Materials and Methods

### 2.1. Study Design and Population

**Figure 1:**
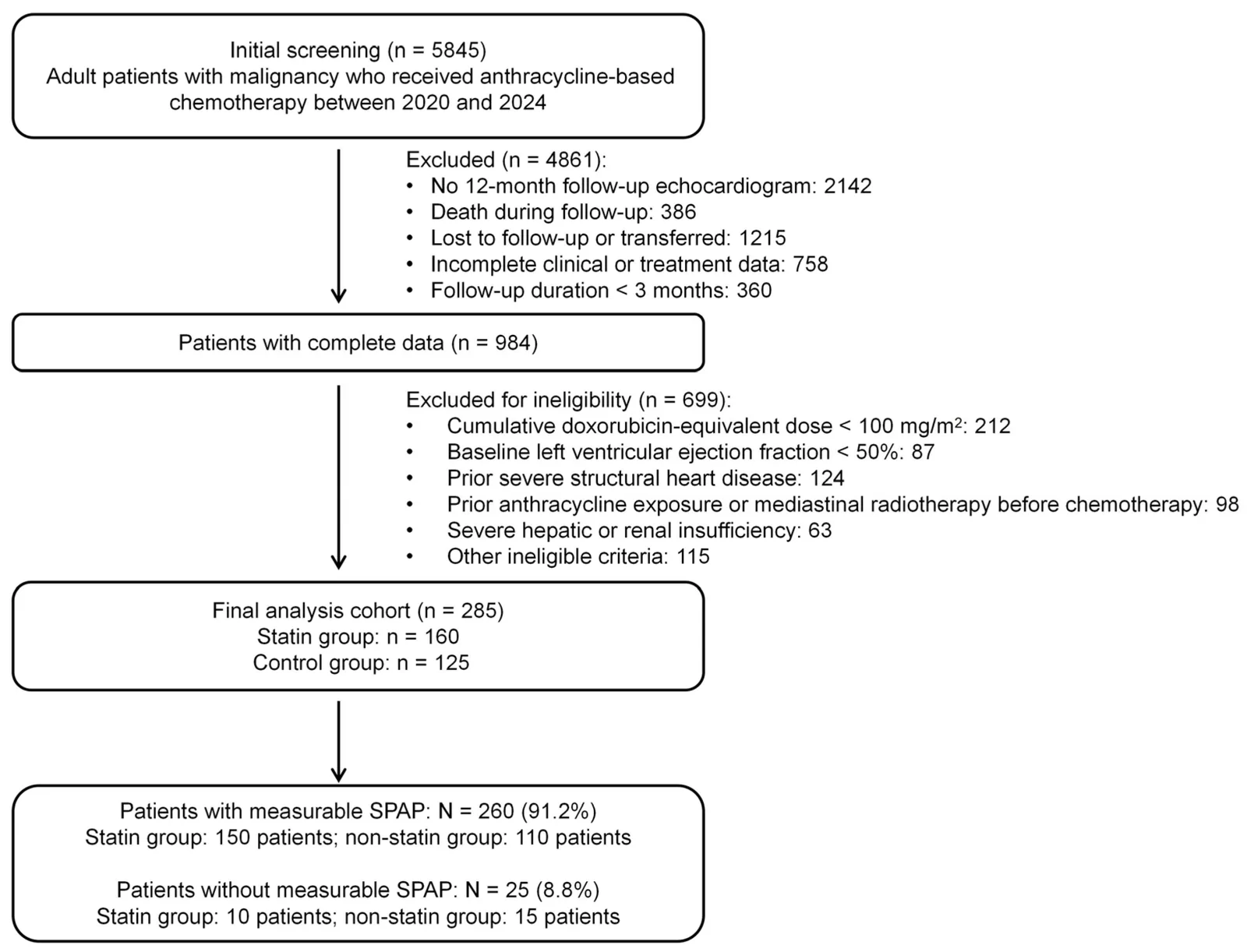
Patient selection flowchart. The flowchart shows the participant selection process from initial screening to the final analytic cohort. Reasons for exclusion at each stage are indicated. The final cohort included 285 patients, of whom 260 had complete serial SPAP measurements available for pulmonary hemodynamic analyses.

This single-center retrospective cohort study consecutively enrolled adult patients aged 18 years or older with histopathologically confirmed malignancies. All patients received anthracycline-containing chemotherapy at the First Affiliated Hospital of USTC between January 2020 and December 2024. We initially screened 5845 patients. A total of 4861 patients were excluded at the initial screening stage. The reasons included absence of the required 12-month follow-up echocardiogram in 2142 patients, death during follow-up in 386 patients, loss to follow-up or transfer to another hospital in 1215 patients, incomplete clinical or treatment data in 758 patients, and insufficient follow-up duration in 360 patients. This left 984 patients with complete baseline and follow-up data for further eligibility assessment. The detailed patient selection process is summarized in Figure 1. Inclusion criteria required a cumulative doxorubicin-equivalent dose (DED) of at least 100 mg/m^2^, baseline transthoracic echocardiography (TTE) within 1 month before chemotherapy, follow-up TTE at 12 ± 1 months after chemotherapy, and complete medical records. Exclusion criteria comprised pre-existing severe structural heart disease. This included heart failure, myocardial infarction, severe valvular heart disease, or complex congenital heart disease. Additional exclusion criteria were baseline LVEF below 50%, prior anthracycline exposure or mediastinal radiotherapy before chemotherapy initiation, and severe hepatic or renal dysfunction. A total of 699 patients were excluded based on these criteria.

The final analytic cohort included 285 patients. Patients who received concurrent or sequential mediastinal radiotherapy during chemotherapy were not excluded. These patients were retained in the final analytic cohort. Mediastinal radiotherapy was adjusted for as a treatment-related covariate in all multivariable models. Follow-up TTE was defined as the examination performed at 12 ± 1 months after the baseline study. The actual interval between the two examinations was recorded for all patients. Cumulative doses of different anthracyclines were converted to DED using the cardiovascular toxicity equivalence ratios recommended by the 2022 ESC Guidelines on Cardio-Oncology [19]. DED was treated as a continuous variable in primary analyses, with a descriptive subgroup threshold of 250 mg/m^2^.

The study was approved by the Ethics Committee of the First Affiliated Hospital of USTC (approval number: [2024-RE-424]) and conducted in accordance with the Declaration of Helsinki. Written informed consent was waived due to the retrospective nature of the study.

### 2.2. Echocardiographic Assessment

All patients underwent comprehensive TTE at baseline and at 12-month follow-up, performed by certified sonographers using GE Vivid E9 (GE Medical Systems Ultrasound & Primary Care Diagnostics, LLC, Wauwatosa, WI, USA) or Philips EPIQ 7C systems (Philips Ultrasound, Inc., Bothell, WA, USA) according to the joint ASE and EACVI guidelines [20]. Baseline and follow-up examinations were performed on the same ultrasound platform in 87% of patients, and the distribution of platform type was balanced between groups (*p* = 0.62), minimizing equipment-related systematic bias. All offline image readers were blinded to statin exposure and clinical outcomes.

Left ventricular end-diastolic and end-systolic volumes were measured using the biplane modified Simpson method and indexed to body surface area to account for differences in body size and sex distribution [21]. The indexed values were denoted as left ventricular end-diastolic volume index (LVEDVi) and left ventricular end-systolic volume index (LVESVi). Diastolic function was assessed in accordance with the 2016 ASE and EACVI guidelines using pulsed-wave Doppler for mitral inflow E wave and E/A ratio and tissue Doppler for mean e’ velocity averaged from the septal and lateral mitral annulus [22]. The E/e’ ratio was calculated as mitral inflow E wave velocity divided by mean annular e’ velocity. Due to the retrospective design, complete parameters for integrated diastolic grading, including left atrial volume index and peak tricuspid regurgitation velocity, were not available for all patients. We therefore report changes in individual diastolic parameters rather than assigning an overall diastolic function grade. Given that E/e’ is strongly influenced by preload conditions, which may vary substantially during chemotherapy due to altered volume status and tumor burden, changes in E/e’ alone are not interpreted as definitive improvement or deterioration in diastolic function.

Systolic pulmonary artery pressure (SPAP) was calculated from tricuspid regurgitation peak velocity using the simplified Bernoulli equation and estimated right atrial pressure (RAP), in accordance with the 2025 ASE recommendations for the echocardiographic assessment of the right heart and pulmonary circulation [23]. Patients with suboptimal tricuspid regurgitation signals were excluded from SPAP-related analyses. Three distinct SPAP-derived variables were defined and are not interchangeable. Signed SPAP change was defined as follow-up value minus baseline value in mmHg, representing the directional change. Absolute SPAP change was defined as the absolute value of the difference between follow-up and baseline measurements in mmHg, representing the magnitude of change irrespective of direction. The relative change amplitude of estimated SPAP was defined as the absolute difference divided by the baseline value multiplied by 100, representing the magnitude of change normalized to baseline pressure. This relative change amplitude of estimated SPAP does not constitute a statistical adjustment for baseline SPAP and may be susceptible to regression to the mean and measurement error. Component analyses comparing TRPG and RAP changes were descriptive only. They compared signed mean changes and did not represent a mathematical decomposition of the absolute-change SPAP endpoint.

Given the retrospective design and incomplete availability of speckle-tracking global longitudinal strain and cardiac biomarkers in this cohort, we used a simplified study-defined LVEF-based cancer therapeutics-related cardiac dysfunction (CTRCD) endpoint aligned with the core LVEF criteria of the 2022 ESC Guidelines on Cardio-Oncology and the 2025 Chinese Expert Consensus [19,24]. Asymptomatic CTRCD was defined as an absolute LVEF decrease of at least 10% from baseline to below 50%. Symptomatic CTRCD was defined as an absolute LVEF decrease of at least 5% from baseline with concurrent symptoms or signs of heart failure. This simplified endpoint does not incorporate global longitudinal strain or biomarker criteria for mild asymptomatic CTRCD, as specified in contemporary integrated definitions, which should be considered when interpreting the low event rate.

For quality control, 30 randomly selected echocardiographic studies were remeasured by two independent blinded sonographers. Parameter-specific intraclass correlation coefficients with 95% confidence intervals were calculated to evaluate both intra- and inter-observer reproducibility for all reported echocardiographic parameters (Supplementary Table S3). All parameters showed good to excellent reproducibility, with inter-observer intraclass correlation coefficients ranging from 0.83 to 0.92 and intra-observer intraclass correlation coefficients ranging from 0.85 to 0.94. Bland–Altman analysis for estimated SPAP showed a mean difference of 0.3 mmHg with 95% limits of agreement from −2.07 to 2.67 mmHg. The width of these limits of agreement exceeds the observed between-group difference in absolute SPAP change. This emphasizes that the clinical meaning of such a small between-group difference remains uncertain.

### 2.3. Covariate Definitions

Baseline clinical characteristics and treatment data were extracted from the hospital electronic medical record system, including demographic factors such as age, sex, and body mass index (BMI); tumor type distribution; cardiovascular comorbidities including hypertension, diabetes, and dyslipidemia; cumulative anthracycline dose; concurrent medications including trastuzumab and dexrazoxane; and mediastinal radiotherapy history. Statin exposure was defined as regular statin therapy initiated before or during chemotherapy with a continuous duration of at least 3 months, verified by outpatient and discharge electronic prescription records. Dyslipidemia was diagnosed according to the Chinese Guidelines for the Management of Dyslipidemia in Adults, defined as abnormal lipid levels including total cholesterol of at least 6.2 mmol/L, low-density lipoprotein cholesterol of at least 4.2 mmol/L, triglycerides of at least 2.26 mmol/L, high-density lipoprotein cholesterol below 1.04 mmol/L, or a pre-existing diagnosis with ongoing lipid-lowering therapy.

### 2.4. Statistical Analysis

Statistical analyses were performed using SPSS 26.0 and R 4.3.2 software. Continuous and categorical variables were compared via *t*-tests and chi-square or Fisher exact tests.

Endpoints were categorized as primary, secondary, exploratory, and descriptive for the present analysis. The primary endpoint was change in LVEF from baseline to 12 months. Secondary endpoints included changes in LVESVi and mean e’ velocity from baseline to 12 months. Exploratory endpoints included changes in LVEDVi, E/A ratio, E/e’ ratio, and all SPAP-derived variables, as well as subgroup analyses, restricted cubic spline (RCS) dose–response analyses, and exploratory association decomposition analysis. The incidence of study-defined CTRCD was treated as a descriptive endpoint only, given the expected low event rate and limited statistical power. With the exception of the primary endpoint, all analyses were exploratory in nature, and no formal correction for multiple testing was applied. Accordingly, findings from secondary and exploratory endpoints should be interpreted as hypothesis-generating. Sensitivity analyses were conducted to evaluate the robustness of the primary findings.

For SPAP-related analyses, the primary exploratory model used the relative change amplitude of estimated SPAP as the dependent variable. Two additional sensitivity analyses were performed. The first was a conventional analysis of covariance (ANCOVA) model with follow-up SPAP as the dependent variable, adjusted for baseline SPAP and all covariates. The second used absolute SPAP change in mmHg as the dependent variable, adjusted for all covariates. The primary SPAP-related analyses used a complete-case approach, restricted to the 260 patients with analyzable tricuspid regurgitation signals at both baseline and 12-month follow-up. Twenty-five patients were excluded from SPAP analyses due to suboptimal or non-detectable tricuspid regurgitation signals at either time point. To assess potential selection bias, we compared baseline demographic, clinical, and echocardiographic characteristics between patients with and without complete serial SPAP measurements. This comparison is descriptive only. The absence of significant baseline differences does not formally establish a missing-at-random mechanism, nor does it exclude non-random missingness related to unmeasured factors such as body habitus, pulmonary disease, or right ventricular function. As a sensitivity analysis, multiple imputation by chained equations was performed using all baseline demographic, clinical, treatment, and echocardiographic covariates, including all 285 patients. This analysis assesses the robustness of SPAP-related findings under the assumptions of the specified imputation model. It does not exclude bias from a missing-not-at-random mechanism (Supplementary Table S5). For non-negative SPAP change variables including absolute change and relative change amplitude of estimated SPAP, regression diagnostics were performed to assess linearity, normality of residuals, and homoscedasticity. The primary analyses used conventional multivariable linear regression with ordinary standard errors. HC3 robust standard errors and 1000-resample bootstrap inference were used as sensitivity analyses to confirm the stability of effect estimates, as these variables may be asymmetrically distributed. Results from these sensitivity analyses were consistent with the primary findings and are not reported separately.

Multivariable linear regression models evaluated the independent association between statin use and echocardiographic changes. Covariates were selected a priori based on clinical relevance, prior literature, and directed acyclic graph (DAG) principles, including demographic factors such as age, sex, and BMI; cardiovascular comorbidities including hypertension, diabetes, and dyslipidemia; cancer-treatment-related factors including cumulative anthracycline equivalent dose, dexrazoxane use, trastuzumab use, and mediastinal radiotherapy; and the baseline value of the corresponding outcome parameter to control for regression to the mean. Cumulative anthracycline equivalent dose was used as the primary measure of chemotherapy exposure, as it accounts for differences in dose intensity across regimens and is more clinically meaningful than cycle count alone. Variables including tumor stage, detailed smoking history, estimated glomerular filtration rate (eGFR), and specific chemotherapy regimen details were not uniformly available in retrospective records and thus not included in the primary model [25]. Three sensitivity analyses were conducted by sequentially excluding patients receiving dexrazoxane, trastuzumab, or mediastinal radiotherapy.

Subgroup analyses were conducted stratified by trastuzumab use, mediastinal radiotherapy, and anthracycline dose below versus at least 250 mg/m^2^, with interaction terms added to regression models to test for effect modification. Propensity score matching (PSM) was applied to reduce selection bias from baseline imbalances, with the target estimand being the average treatment effect in the treated. Given the clinically important baseline differences in age, sex, and multiple echocardiographic parameters between groups, propensity scores were calculated via logistic regression with statin use as the dependent variable and all baseline covariates as independent variables, including all baseline echocardiographic parameters (LVEF, LVEDVi, LVESVi, mean e’ velocity, and estimated SPAP). One-to-one nearest-neighbor matching was performed without replacement, with a caliper width set at 0.2 standard deviations of the logit of the propensity score [26]. Adequate overlap of propensity scores between groups was confirmed visually. Covariate balance before and after matching was evaluated via standardized mean differences (SMDs), with a value below 0.1 indicating adequate balance. SMDs for every variable entered into the propensity score model are reported in Supplementary Table S1. A Love plot containing the same full covariate set was used to graphically display balance (Supplementary Figure S1). Matched analyses accounted for the paired structure of the data using linear regression models with cluster-robust standard errors at the matched-pair level. Residual confounding cannot be fully excluded even after matching.

RCS regression with 4 knots was used to explore potential nonlinearity in the association between cumulative anthracycline dose and LVEF change, stratified by statin use. Formal tests for nonlinearity and group-by-dose interaction were performed. An exploratory association decomposition analysis was conducted to examine the extent to which the relative change amplitude of estimated SPAP co-varies with the statistical association between statin use and LVEF change. Bias-corrected and accelerated (BCa) bootstrap with 1000 resamples was used to calculate 95% confidence intervals. Given that both the SPAP-derived variable and LVEF outcome were measured at the same 12-month follow-up time point, this analysis describes only a statistical association structure and does not establish a biological mediation pathway, temporal precedence, or causal inference. The result is strictly hypothesis-generating [27].

## 3. Results

### 3.1. Baseline Characteristics

A total of 5845 consecutive anthracycline-treated patients were initially screened. The participant selection process is summarized in Figure 1. The final analytic cohort included 285 patients, comprising 160 statin users and 125 non-statin users. The actual interval between baseline and follow-up transthoracic echocardiography was 12.8 ± 0.9 months in the statin group and 12.9 ± 0.8 months in the non-statin group, with no significant between-group difference (*p* = 0.32). SPAP measurements were available for 260 of 285 patients (91.2%). Twenty-five patients were excluded from SPAP-related analyses due to suboptimal tricuspid regurgitation signals at either time point. Comparison of baseline characteristics between patients with and without complete serial SPAP measurements revealed no significant differences in age, sex, body mass index, cardiovascular comorbidities, cumulative anthracycline dose, or baseline left ventricular parameters (all *p* > 0.05). This descriptive comparison suggests no overt baseline selection bias but does not formally confirm a missing-at-random mechanism.

Baseline characteristics of the final cohort are summarized in Table 1. The two groups were well balanced for most parameters. Body mass index; cardiovascular comorbidities; tumor-type distribution (*p* > 0.05); cumulative doxorubicin-equivalent dose (282.2 ± 73.2 vs. 274.2 ± 62.2 mg/m^2^, *p* = 0.3); rates of dexrazoxane use, trastuzumab use, and mediastinal radiotherapy; baseline tricuspid regurgitant pressure gradient (TRPG); and baseline SPAP all showed no significant difference (all *p* > 0.05). Several baseline differences were observed and adjusted for in all multivariable and propensity score models. These differences were not only statistically significant but also potentially clinically important. Patients in the statin group were older (58.1 ± 12.0 vs. 54.6 ± 9.3 years, *p* = 0.006) and included a higher proportion of women (88.8% vs. 76.8%, *p* = 0.010). For echocardiographic parameters, the statin group had lower baseline LVEF (66.5 ± 5.6% vs. 68.8 ± 5.5%, *p* < 0.001), higher baseline LVEDVi (70.8 ± 12.7 vs. 66.7 ± 11.8 mL/m^2^, *p* = 0.005), higher baseline LVESVi (23.8 ± 6.1 vs. 20.7 ± 5.0 mL/m^2^, *p* < 0.001), higher baseline RAP (5.3 ± 2.2 vs. 4.3 ± 2.0 mmHg, *p* < 0.001), and lower mean e’ velocity (9.5 ± 1.6 vs. 10.5 ± 1.3 cm/s, *p* < 0.001). These baseline differences may independently influence subsequent cardiac function changes and were therefore included as covariates in all regression models and in the PSM model.

**Table 1:** Baseline characteristics of the study cohort.

Characteristic	Total (*n* = 285) ^1^	Non-Statin (*n* = 125) ^1^	Statin (*n* = 160) ^1^	*p* Value ^2^
Age, years	56.5 ± 11.0	54.6 ± 9.3	58.1 ± 12.0	0.006
Sex				0.010
Female	238 (84%)	96 (77%)	142 (89%)	
Male	47 (16%)	29 (23%)	18 (11%)	
Body mass index, kg/m^2^	23.8 ± 4.4	24.3 ± 3.9	23.5 ± 4.8	0.11
Hypertension	40 (14%)	19 (15%)	21 (13%)	0.7
Diabetes mellitus	29 (10%)	16 (13%)	13 (8%)	0.2
Dyslipidemia	44 (15%)	24 (19%)	20 (13%)	0.14
Cumulative doxorubicin-equivalent dose, mg/m^2^	278.7 ± 68.6	274.2 ± 62.2	282.2 ± 73.2	0.3
Trastuzumab co-administration	51 (18%)	22 (18%)	29 (18%)	>0.9
Dexrazoxane use	82 (29%)	39 (31%)	43 (27%)	0.4
Mediastinal radiotherapy	39 (14%)	21 (17%)	18 (11%)	0.2
Tumor characteristics				
Tumor type				0.973
Breast cancer	143(50.0%)	62 (49.6%)	81 (50.6%)	
Lymphoma	118(41.4%)	52 (50.6%)	66 (41.2%)	
Other malignancies	24(8.4%)	11 (8.8%)	13 (8.1%)	

^1^ Mean ± SD; *n* (%). ^2^ Welch two-sample *t*-test; Fisher’s exact test. Continuous variables are presented as mean ± standard deviation and compared using independent samples *t*-tests. Categorical variables are presented as count (percentage) and compared using chi-square tests or Fisher’s exact tests as appropriate. Baseline echocardiographic parameters are reported separately in Table 2 to avoid duplication.

**Table 2:** Echocardiographic parameters at baseline and 12-month follow-up.

Parameter	Non-Statin (*n* = 125) ^1^	Statin (*n* = 160) ^1^	*p* Value ^2^
Baseline LVEF, %	68.8 ± 5.5	66.5 ± 5.6	<0.001
12-month LVEF, %	64.5 ± 4.6	64.9 ± 6.2	0.5
Change in LVEF, %	−4.3 ± 7.0	−1.6 ± 8.5	0.004
Baseline LVEDVi, mL/m^2^	66.7 ± 11.8	70.8 ± 12.7	0.005
12-month LVEDVi, mL/m^2^	70.2 ± 9.8	72.6 ± 13.1	0.071
Change in LVEDVi, mL/m^2^	3.4 ± 12.2	1.8 ± 11.0	0.2
Baseline LVESVi, mL/m^2^	20.7 ± 5.0	23.8 ± 6.1	<0.001
12-month LVESVi, mL/m^2^	24.9 ± 4.6	25.2 ± 5.3	0.6
Change in LVESVi, mL/m^2^	4.2 ± 6.0	1.5 ± 6.2	<0.001
Baseline mean e’, cm/s	10.5 ± 1.3	9.5 ± 1.6	<0.001
12-month mean e’, cm/s	9.1 ± 1.7	9.1 ± 1.6	0.7
Change in mean e’, cm/s	−1.4 ± 1.7	−0.4 ± 1.1	<0.001
Baseline mitral E wave, cm/s	77.0 ± 10.7	78.7 ± 12.3	0.2
12-month mitral E wave, cm/s	59.7 ± 5.0	64.6 ± 12.0	<0.001
Change in mitral E wave, cm/s	−17.4 ± 11.1	−14.0 ± 15.3	0.035
Baseline E/A ratio	0.9 ± 0.1	0.9 ± 0.2	0.040
12-month E/A ratio	0.7 ± 0.1	0.8 ± 0.3	0.008
Change in E/A ratio	−0.2 ± 0.2	−0.2 ± 0.3	0.5
Baseline E/e’ ratio	7.5 ± 1.5	8.5 ± 1.8	<0.001
12-month E/e’ ratio	6.8 ± 1.3	7.3 ± 1.7	0.007
Change in E/e’ ratio	−0.7 ± 1.7	−1.2 ± 2.0	0.022
Baseline SPAP, mmHg	26.7 ± 3.2	27.3 ± 3.3	0.2
Unknown	15	10	
12-month SPAP, mmHg	30.3 ± 6.1	30.6 ± 4.9	0.7
Unknown	15	10	
Signed change in SPAP, mmHg	3.6 ± 5.7	3.3 ± 4.0	0.6
Unknown	15	10	
Absolute change in SPAP, mmHg	5.8 ± 3.3	4.6 ± 2.3	0.001
Unknown	15	10	
Relative change amplitude of estimated SPAP, %	22.3 ± 13.3	17.3 ± 9.4	0.001
Unknown	15	10	
Baseline TRPG, mmHg	22.4 ± 4.4	22.1 ± 4.3	0.6
12-month TRPG, mmHg	25.5 ± 6.9	25.3 ± 5.8	0.8
Change in TRPG, mmHg	3.2 ± 5.6	3.3 ± 4.0	0.9
Baseline RAP, mmHg	4.2 ± 1.9	5.3 ± 2.2	<0.001
Unknown	15	10	
12-month RAP, mmHg	4.4 ± 1.9	5.3 ± 2.1	<0.001
Unknown	15	10	
Change in RAP, mmHg	0.2 ± 0.6	0.1 ± 0.4	0.026
Unknown	15	10	

^1^ Mean ± SD. ^2^ Welch two-sample *t*-test. Continuous variables are presented as mean ± standard deviation. *p* values refer to between-group comparisons of change from baseline using independent samples *t*-tests. For systolic pulmonary artery pressure (SPAP)-related parameters, analyses included 260 patients with complete serial SPAP measurements (150 statin users and 110 non-statin users). Abbreviations: LVEF, left ventricular ejection fraction; LVEDVi, left ventricular end-diastolic volume index; LVESVi, left ventricular end-systolic volume index; TRPG, tricuspid regurgitant pressure gradient; RAP, right atrial pressure.

Quality control testing confirmed good reproducibility for all core echocardiographic parameters. Parameter-specific intraclass correlation coefficients with 95% confidence intervals were 0.92 (0.84 to 0.96) for LVEF, 0.90 (0.81 to 0.95) for LVESVi, 0.88 (0.78 to 0.94) for mean e’ velocity, and 0.86 (0.74 to 0.93) for estimated SPAP, with no meaningful difference between inter- and intra-observer reproducibility.

Using the study-defined LVEF-based CTRCD endpoint, CTRCD occurred in 2 of 160 patients (1.25%) in the statin group and 6 of 125 patients (4.8%) in the non-statin group. The incidence was numerically lower in the statin group, but the difference was not statistically significant (two-sided Fisher exact test, *p* = 0.15; OR = 0.25, 95% CI 0.05 to 1.28). With only eight total events, the study was not powered to detect a statistically significant difference in this clinical endpoint. This finding should therefore be interpreted as a descriptive observation only. It does not provide evidence of an association between statin use and reduced clinical CTRCD risk.

### 3.2. Primary Outcomes

Univariate comparisons of cardiac parameter changes between groups at 12 months are presented in Table 2. For left ventricular systolic parameters, the statin group showed a smaller reduction in LVEF (−1.6 ± 8.5% vs. −4.3 ± 7.0%, *p* = 0.004) and less LVESVi dilation (1.5 ± 6.2 vs. 4.2 ± 6.0 mL/m^2^, *p* < 0.001) compared with the non-statin group. No significant between-group difference was observed for LVEDVi change (1.8 ± 11.0 vs. 3.4 ± 12.2 mL/m^2^, *p* = 0.2). Notably, the two groups had significant baseline differences in LVEF, LVEDVi, and LVESVi (all *p* < 0.01), which may confound these univariate associations.

For diastolic parameters, the statin group had a milder decline in mean e’ velocity (−0.4 ± 1.1 vs. −1.4 ± 1.7 cm/s, *p* < 0.001) and a smaller reduction in mitral E wave velocity (−14.0 ± 15.3 vs. −17.4 ± 11.1 cm/s, *p* = 0.035). The 12-month E/A ratio was higher in the statin group (0.8 ± 0.3 vs. 0.7 ± 0.1, *p* = 0.008), but the change in E/A ratio from baseline did not differ significantly between groups (−0.2 ± 0.3 vs. −0.2 ± 0.2, *p* = 0.5). Although the statin group demonstrated a significantly greater reduction in the E/e’ ratio from baseline in the univariate analysis (−1.2 ± 2.0 vs. −0.7 ± 1.7, *p* = 0.022), this between-group difference lost statistical significance following multivariable adjustment for baseline E/e’ and other confounders (*β* = 0.19, 95% CI −0.18 to 0.57, *p* = 0.312). The E/A ratio, mitral E wave velocity, and E/e’ ratio are highly load-dependent. Their variations during chemotherapy may be confounded by fluctuating volume status and tumor burden. These changes therefore cannot be interpreted as definitive evidence of improved diastolic function. In contrast, the attenuated decline in e’ velocity, a relatively load-independent marker of early myocardial relaxation, suggests that statin therapy may be associated with the preservation of early diastolic relaxation.

For pulmonary hemodynamic parameters, no significant difference was observed in signed change in estimated SPAP (3.3 ± 4.0 vs. 3.6 ± 5.7 mmHg, *p* = 0.6). However, the statin group had a smaller absolute SPAP change (4.6 ± 2.3 vs. 5.8 ± 3.3 mmHg, *p* = 0.001) and a lower relative change amplitude of estimated SPAP (17.3 ± 9.4% vs. 22.3 ± 13.3%, *p* = 0.001). Component analysis showed that the change in TRPG did not differ between groups (3.2 ± 4.0 vs. 3.2 ± 5.6 mmHg, *p* = 0.868), whereas the change in estimated RAP was smaller in the statin group (0.1 ± 0.4 vs. 0.2 ± 0.6 mmHg, *p* = 0.021). The distribution of relative change amplitude of estimated SPAP is summarized in Supplementary Table S2.

Given the significant baseline imbalances between groups in age, sex, baseline LVEF, LVESVi, mean e’ velocity, and RAP, we further performed multivariable linear regression adjusted for these clinically relevant confounders and the baseline value of the corresponding outcome parameter (Table 3). After adjustment, statin use remained independently associated with diastolic parameters, including a milder decline in mean e’ velocity (*β* = 0.75 cm/s, 95% CI 0.41 to 1.09, *p* < 0.001) and a smaller reduction in E/A ratio (*β* = 0.06, 95% CI 0.01 to 0.11, *p* = 0.02). However, the associations with LVEF change (*β* = 0.28%, 95% CI −1.11 to 1.66, *p* = 0.694) and LVESVi change (*β* = −0.87 mL/m^2^, 95% CI −1.98 to 0.24, *p* = 0.124) were attenuated after multivariable adjustment and were no longer statistically significant. This pattern is compatible with confounding by baseline differences in age, sex, and baseline cardiac parameters.

For pulmonary hemodynamics in the 260-patient complete case–cohort, signed change in estimated SPAP did not differ significantly between groups after adjustment (*β* = −0.14 mmHg, 95% CI −1.39 to 1.11, *p* = 0.821). The adjusted R^2^ for this signed SPAP change model was −0.011, indicating poor explanatory performance after accounting for model complexity. This should not be interpreted as evidence that the crude association was entirely attributable to covariates, nor that absolute SPAP change is not a meaningful pharmacological target. In exploratory analyses, both absolute SPAP change (*β* = −1.25 mmHg, 95% CI −1.97 to −0.53, *p* < 0.001) and relative change amplitude of estimated SPAP (*β* = −4.55%, 95% CI −7.34 to −1.76, *p* = 0.001) were significantly lower in the statin group. Results remained consistent in sensitivity analyses using HC3 robust standard errors and 1000-resample bootstrap inference, as well as in a conventional ANCOVA model with follow-up SPAP adjusted for baseline SPAP. Component analysis of estimated SPAP (Supplementary Table S4) showed that the signed mean change in estimated RAP was smaller in the statin group (0.1 ± 0.4 vs. 0.2 ± 0.6 mmHg, *p* = 0.021). The signed mean change in tricuspid regurgitant pressure gradient did not differ between groups (3.2 ± 4.0 vs. 3.2 ± 5.6 mmHg, *p* = 0.868). This component analysis compares signed mean changes only. It cannot mathematically decompose the absolute-change SPAP endpoint. The between-group difference in mean RAP change is only approximately 0.1 mmHg. This is substantially smaller than the observed between-group difference in absolute SPAP change. The dispersion of TRPG changes also differs between groups. These findings should therefore be interpreted as descriptive only. They should not be taken as evidence that one component specifically accounts for the difference in absolute SPAP change magnitude. All component values should also be interpreted in the context of the measurement error inherent to Doppler-derived pulmonary pressure estimates. The estimated association between statin use and relative change amplitude of estimated SPAP was similar in the multiple-imputation sensitivity analysis including all 285 patients (*β* = −4.49%, 95% CI −7.27 to −1.71, *p* = 0.002, Supplementary Table S5). This analysis evaluates robustness under the specified imputation model. It does not exclude potential bias arising from a missing-not-at-random mechanism.

### 3.3. Sensitivity and Subgroup Analyses

To verify the robustness of the primary findings, we performed three exclusion-based sensitivity analyses (Table 4), sequentially excluding patients receiving dexrazoxane, trastuzumab, or mediastinal radiotherapy. In all three sensitivity cohorts, statin use remained significantly associated with milder mean e’ decline (all *p* < 0.001) and lower relative change amplitude of estimated SPAP (all *p* < 0.05). The direction of effect for LVEF change and LVESVi change was consistent with the primary analysis, but neither parameter reached statistical significance in any sensitivity cohort. PSM analysis was performed separately to further address baseline imbalances, and the results are presented in Section 3.4.

We further conducted subgroup analyses to explore potential effect modification by clinical characteristics (Table 5), stratified by trastuzumab use, mediastinal radiotherapy, and cumulative doxorubicin-equivalent dose (below vs. at least 250 mg/m^2^, the clinically recognized higher-risk threshold). No significant effect modification was detected for any stratification variable (all *p* for interaction > 0.05), indicating no statistically significant heterogeneity in the association between statin use and LVEF change across subgroups. The direction of association was consistent across most subgroups, but no subgroup showed a statistically significant association with LVEF change. A borderline association was observed in the mediastinal radiotherapy subgroup (*β* = 3.27%, 95% CI −0.13 to 6.67, *p* = 0.059), but the corresponding interaction test was not significant (*p* for interaction = 0.06). This finding should be interpreted as a chance observation likely related to the small sample size of this subgroup (*n* = 39). The lack of significant associations in smaller subgroups is likely attributable to limited statistical power.

### 3.4. Propensity-Score-Matching Analysis

To further reduce potential selection bias from baseline imbalances, we performed 1:1 nearest-neighbor PSM without replacement, with a caliper width of 0.2 standard deviations of the logit of the propensity score, targeting the average treatment effect in the treated. Propensity scores were estimated via logistic regression with statin use as the dependent variable and all baseline covariates as independent variables. Adequate overlap of propensity scores between groups was confirmed visually. A total of 72 matched pairs were generated. All baseline echocardiographic parameters were included in the propensity score model to account for the clinically important baseline differences in cardiac structure and function. A total of 88 statin users and 53 non-statin users remained unmatched. Matching improved overall covariate balance. All five baseline echocardiographic parameters achieved SMD below 0.10 after matching. Three clinical covariates retained residual SMDs slightly above 0.10 after matching. These were age, hypertension, and cardioprotective agent use. These variables were retained in all post-matching regression models to adjust for residual imbalance. The matched analysis pertains to this selected matched population and may not generalize to the full cohort. Post-matching multivariable regression, accounting for the matched structure using cluster-robust standard errors at the matched-pair level, showed that statin use remained significantly associated with milder mean e’ decline (*β* = 0.84 cm/s, 95% CI 0.35 to 1.33, *p* < 0.001). All 72 matched pairs (144 patients) had complete serial SPAP measurements. In this matched cohort, statin use was associated with a lower relative change amplitude of estimated SPAP (*β* = −5.67%, 95% CI −9.37 to −1.97, *p* = 0.003). After adjustment for residual imbalance, statin use was also associated with less LVESVi dilation (*β* = −1.78 mL/m^2^, 95% CI −3.16 to −0.40, *p* = 0.013). The effect size for this parameter was modest. This association was not observed in the primary multivariable analysis or the three exclusion-based sensitivity cohorts. No significant association was observed for LVEF change (*β* = 0.95%, 95% CI −1.03 to 2.93, *p* = 0.349).

### 3.5. Dose–Response and Exploratory Association Decomposition Analysis

**Figure 2:**
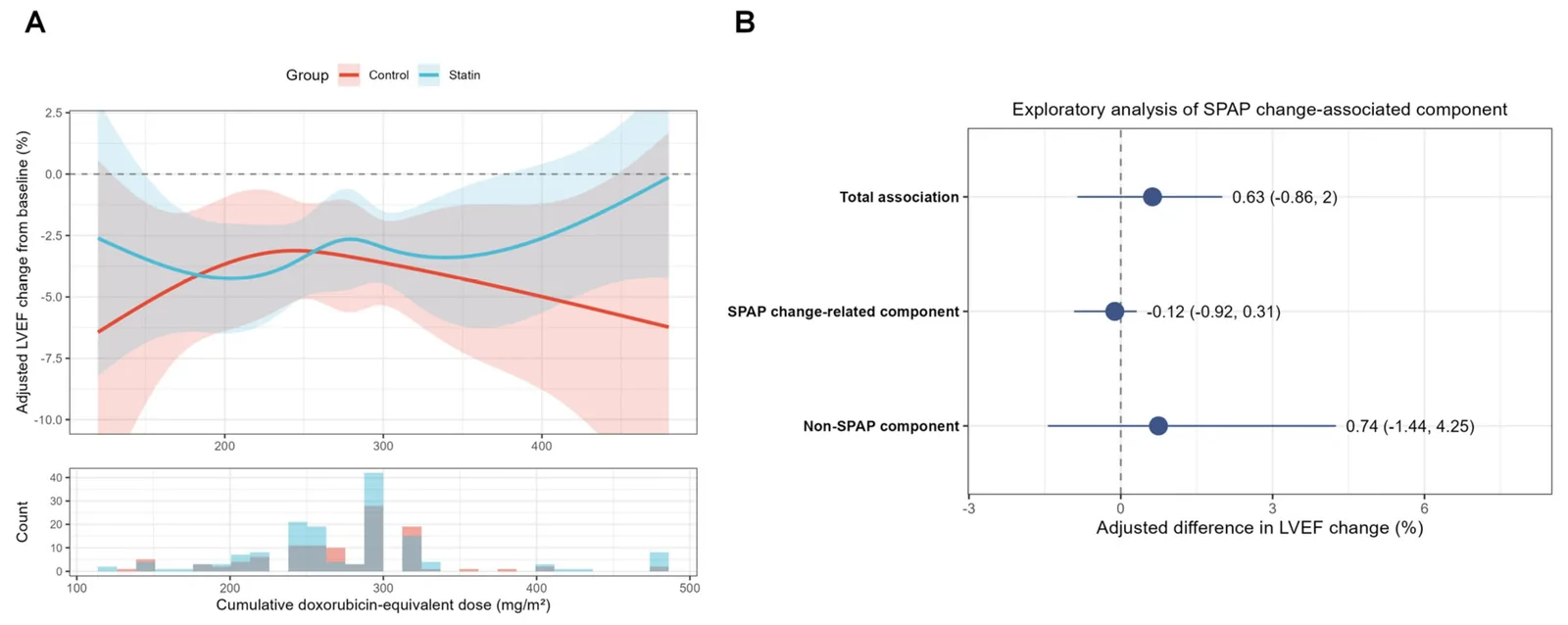
Dose–response relationship and exploratory association decomposition analysis. (**A**) RCS analysis of the association between cumulative doxorubicin-equivalent dose and adjusted change in LVEF from baseline, stratified by statin use. Red = Control; blue = Statin. Shaded areas represent 95% confidence intervals. The histogram below shows the distribution of cumulative doxorubicin-equivalent dose in the two groups. *p* for nonlinearity was 0.861, and *p* for group-by-dose interaction was 0.72. (**B**) Exploratory association decomposition analysis examining the co-variation between relative change amplitude of estimated SPAP and the association between statin use and LVEF change. Points represent adjusted *β* coefficients, and horizontal lines represent 95% confidence intervals. The vertical dashed line indicates the null value. Analyses included 260 patients with complete concurrent SPAP and LVEF data. All three components were not statistically significant, and this analysis describes only a correlational structure without causal interpretation.

RCS regression with four knots placed at the 5th, 35th, 65th, and 95th percentiles of the cumulative doxorubicin-equivalent dose showed no significant nonlinear association between cumulative anthracycline dose and LVEF change (*p* for nonlinearity = 0.861, Figure 2A). The dose–response curves for the statin and non-statin groups largely overlapped across the observed dose range, with no statistically significant between-group separation at any dose level. Apparent curve fluctuation at doses above 400 mg/m^2^ was attributable to the small number of patients in this high-dose range and should not be interpreted as a dose-related effect or evidence of greater benefit at higher doses. Formal testing for group-by-dose interaction was not significant (*p* = 0.72), consistent with the subgroup analysis findings of no significant effect modification.

Among the 260 patients with complete concurrent SPAP and LVEF data, we conducted an exploratory association decomposition analysis to examine the extent to which relative change amplitude of estimated SPAP co-varies with the association between statin use and LVEF change (Figure 2B). After full adjustment for baseline covariates and baseline LVEF, the total association of statin use with LVEF change was not statistically significant (*β* = 0.33%, 95% CI −1.03 to 1.63). Neither the SPAP change-related component (*β* = −0.07%, 95% CI −0.66 to 0.26) nor the non-SPAP component (*β* = 0.41%, 95% CI −1.90 to 3.40) reached statistical significance.

Given that both the SPAP-derived variable and LVEF outcome were measured at the same 12-month follow-up, this analysis describes only a correlational co-variation structure. It cannot satisfy the temporal precedence assumption required for causal mediation, nor can it exclude reverse causation such as secondary pulmonary hypertension due to left ventricular dysfunction or common upstream determinants affecting both pulmonary vasculature and myocardium. The result is strictly hypothesis-generating and should not be interpreted as evidence of a biological mediation pathway.

## 4. Discussion

To our knowledge, this study is among the first to report an association between statin co-administration and smaller relative change amplitude of estimated SPAP during follow-up, without a significant effect on signed mean SPAP change. The component analysis and association decomposition analysis are descriptive only. They cannot establish which hemodynamic component drives the observed difference or define the direction of the relationship between pulmonary pressure and left ventricular function. Both classical left-to-right pathways and right-to-left ventricular interdependence may contribute to the observed co-variation. The direction of this relationship cannot be determined from the present data. All findings related to pulmonary hemodynamics should be interpreted cautiously given the measurement error inherent to Doppler-derived SPAP estimates.

Statin therapy in the context of anthracycline-induced cardiotoxicity risk has attracted growing investigation in cardio-oncology, yet published findings remain inconsistent. Observational real-world studies have consistently reported an association between statin use and lower CTRCD risk as well as smaller LVEF decline. A 2025 conference abstract of six randomized controlled trials including 918 patients reported a 59% reduction in clinical cardiotoxicity events with statin therapy (OR 0.41, 95% CI 0.27 to 0.63), a finding that aligns directionally with our descriptive CTRCD trend. This result has not yet been published as a full-length peer-reviewed systematic review, and its evidentiary weight should be interpreted accordingly. Overall, the published literature on statin therapy in this setting is highly heterogeneous. Observational studies have consistently reported positive associations, while randomized trial results have been mixed, with no uniform demonstration of a cardioprotective effect across all populations and endpoints [28]. In contrast, two recently published multicenter randomized controlled trials, SPARE-HF and PREVENT, failed to demonstrate an association between statin use and LVEF decline. SPARE-HF assessed early cardiac changes at three to four weeks after chemotherapy completion [16]. PREVENT used serial cardiac magnetic resonance imaging with follow-up at 6 and 24 months and also did not show a significant effect of statin therapy on left ventricular systolic function [17]. Our finding of no significant independent association with LVEF change is consistent with the neutral results of these two trials across different follow-up durations.

The observed association with diastolic e’ velocity suggests that statin use may coincide with smaller changes in earlier, more sensitive markers of myocardial injury before overt systolic dysfunction develops. This observation is biologically consistent with preclinical evidence of statin effects on myocardial relaxation and endothelial function [29]. Statins may improve early diastolic relaxation through reduction in myocardial oxidative stress and interstitial fibrosis, both of which contribute to increased ventricular stiffness after anthracycline exposure [30]. The absence of a significant association with systolic LVEF change may reflect the relative insensitivity of LVEF as an early marker of myocardial injury. Diastolic abnormalities typically precede systolic dysfunction in anthracycline-related cardiac dysfunction, and tissue Doppler e’ velocity may detect subtle changes before LVEF declines become apparent [31]. The discordance between the significant e’ finding and the non-significant adjusted E/e’ finding further underscores the importance of interpreting diastolic parameters in the context of loading conditions during chemotherapy. This is consistent with the 2025 ASE/EACVI guideline for the evaluation of left ventricular diastolic function [32]. The discordance between observational studies and randomized trials may reflect several factors beyond follow-up duration. Observational cohorts often include patients with higher baseline cardiovascular risk and longer statin exposure, which may amplify detectable associations with early diastolic markers. Randomized trials typically select lower-risk patients and use more standardized treatment protocols, potentially reducing the magnitude of observable changes. The two study designs also differ in their ability to detect subtle, subclinical functional alterations. Rather than viewing these findings as contradictory, we interpret them as complementary. Randomized trials provide rigorous evidence on systolic function endpoints, while real-world cohort studies like ours offer insights into earlier, more sensitive diastolic and hemodynamic parameters in routine clinical practice.

Anthracycline-induced cardiotoxicity is not confined to the left ventricle. Right heart and pulmonary circulatory injury represent an increasingly recognized dimension of this toxicity in preclinical and clinical studies. Anthracyclines may directly affect pulmonary vascular endothelial cells through oxidative stress and endothelial dysfunction in experimental models [33,34]. A 2024 meta-analysis reported that subclinical right ventricular function declines significantly after anthracycline chemotherapy, independent of LVEF changes. This suggests that right ventricular injury may represent a direct toxic effect rather than a secondary consequence of left ventricular dysfunction [35]. In the present study, we did not directly measure dedicated right ventricular functional parameters such as tricuspid annular plane systolic excursion (TAPSE), S’, or right ventricular fractional area change (RV FAC). Our SPAP findings can therefore only describe an association with right ventricular pressure load. They do not demonstrate effects on right ventricular systolic function or pulmonary vascular resistance. Whether the observed reduction in relative change amplitude of SPAP translates to preserved right ventricular function or improved right ventricular–pulmonary artery (RV-PA) coupling remains entirely unknown and requires dedicated right ventricular imaging in future studies [36].

All mechanistic interpretations discussed below are derived from experimental studies in pulmonary hypertension populations and are purely hypothetical. They were not directly tested in the present clinical cohort, in which mean estimated SPAP values remained within the normal range and pulmonary hypertension was not specifically phenotyped. In established pulmonary arterial hypertension or chronic lung-disease-associated pulmonary hypertension, statins have been shown to modulate endothelial vasomotor tone rather than lower baseline pressure. The RhoA and Rho-kinase pathway, which is inhibited by statins, plays a key role in pulmonary vascular smooth muscle contraction and endothelial permeability in experimental models [37]. Modulation of this pathway could theoretically reduce exaggerated pressure responses to chemotherapy-induced endothelial injury, resulting in smaller changes between measurements. The component analysis showing smaller RAP change rather than tricuspid gradient change suggests that statin effects may be more pronounced on systemic venous tone and right heart-filling dynamics than on pulmonary vascular resistance itself. These mechanistic interpretations are strictly hypothesis-generating, as no molecular, invasive hemodynamic, or dedicated right ventricular functional measurements were performed in this study.

It is biologically plausible that larger changes in pulmonary artery pressure impose greater variability in right ventricular afterload, which could potentially affect RV-PA coupling efficiency over time. Statins may attenuate such hemodynamic variability through pleiotropic endothelial effects, including upregulation of endothelial nitric oxide synthase [38], suppression of the RhoA/Rho-kinase signaling pathway, and reduction in oxidative stress-mediated endothelial apoptosis in the pulmonary vasculature. These actions could theoretically stabilize vasomotor tone and buffer chemotherapy-induced endothelial injury, thereby reducing pressure change amplitude without necessarily altering mean pressure levels. None of these pathways were directly evaluated in the present study. The present data do not demonstrate pulmonary vascular stabilization or improved RV-PA coupling. Whether the observed reduction in pressure change magnitude translates to meaningful improvements in right ventricular function or RV-PA coupling remains entirely unknown and represents an important question for future studies. These mechanistic pathways are proposed as hypotheses to guide future research, not as conclusions drawn from the current study.

These observations generate hypotheses for future research. They should be interpreted within the limits of an observational study design. The distinction between relative change amplitude and mean pressure levels suggests that the magnitude of pulmonary hemodynamic change may represent a distinct exploratory marker in anthracycline-treated patients. Such a marker could complement conventional left ventricular monitoring. It may potentially provide an earlier signal of subclinical dysfunction in future studies. Contemporary cardio-oncology guidelines allow consideration of statin initiation for prevention of anthracycline cardiotoxicity in selected patients. This reflects an evolving but not yet definitive evidence base. The present data do not establish that statins should be used as a broadly applicable preventive adjunct in all anthracycline-treated patients. These findings also do not support risk-stratified statin use based on anthracycline dose alone. Prospective studies are required to determine whether these associations translate into clinical benefit. These implications align with the emerging recognition that anthracycline-induced cardiotoxicity extends beyond the left ventricle to involve the right ventricle and pulmonary circulation, whose dysfunction may contribute to global cardiac impairment through ventricular interdependence. Whether and how to incorporate SPAP monitoring and right ventricular assessment into routine surveillance protocols deserves further investigation. The hypothesis-generating nature of these findings underscores the need for prospective validation before any clinical implementation.

Several limitations should be acknowledged. First, as a single-center retrospective cohort study, residual confounding cannot be completely ruled out despite having conducted multivariable adjustment and PSM that included baseline echocardiographic parameters. The statin group had clinically important baseline differences in age, sex, and multiple cardiac parameters, and even after rigorous adjustment and matching, unmeasured or residual confounding may still contribute to the observed associations. Variables including tumor stage, detailed smoking history, renal function, baseline lipid levels, and specific chemotherapy regimen details were not uniformly available and could not be included in the primary models. Second, only two echocardiographic time points were available, limiting our ability to track dynamic cardiac function changes. The relative change amplitude of estimated SPAP calculated from two time points should be interpreted as a measure of change magnitude between two examinations rather than longitudinal variability. Third, statin exposure was insufficiently characterized. We could not reliably separate statin users who initiated therapy before chemotherapy from those who initiated during chemotherapy. Detailed data on statin type, dose, intensity, adherence, treatment duration, and clinical indication were not systematically collected. The lack of specific data about the type, dose, intensity, duration of treatment, and long-term adherence to statin therapy makes it impossible to be able to determine whether changes on echocardiography are related to a certain statin, its cumulative exposure, or even the best choice in index dates for people younger than 60 years. This precludes assessment of differential effects across statin types or the role of confounding by indication. We therefore cannot make claims about optimal statin regimens or whether the observed associations are independent of lipid lowering. Fourth, SPAP was estimated echocardiographically rather than confirmed by right heart catheterization. A complete-case approach was used for primary SPAP-related analyses, excluding 25 patients with suboptimal tricuspid regurgitation signals at either time point. Although no significant differences in baseline characteristics were observed between included and excluded patients, this descriptive comparison cannot establish a missing-at-random mechanism. Failure to obtain a measurable tricuspid regurgitation signal may depend on unmeasured anatomical or physiological characteristics. These include body habitus, pulmonary disease, or right ventricular function. These factors could influence both the probability of missing measurements and the outcomes of interest. Multiple imputation sensitivity analysis yielded consistent results under the specified imputation model. Residual bias related to missing SPAP measurements cannot be excluded. Fifth, dedicated right ventricular systolic functional parameters, including TAPSE, S’, RV FAC, and RV free-wall longitudinal strain, were not routinely recorded in this retrospective cohort. Because estimated SPAP only reflects right ventricular pressure load and is not a direct measure of right ventricular systolic performance, the potential association between pulmonary hemodynamic changes and right ventricular protection remains speculative. Sixth, the exploratory association decomposition analysis is limited by the synchronous measurement of SPAP change and LVEF change at the same follow-up time point, precluding establishment of temporal ordering or biological mediation pathways. Finally, the sample size was modest, limiting statistical power in certain subgroups and for the low-incidence CTRCD endpoint. These results require validation in larger multicenter prospective studies.

## 5. Conclusions

In this retrospective cohort of patients receiving anthracycline chemotherapy, concomitant statin therapy was associated with smaller changes in diastolic parameters and lower relative change amplitude of estimated SPAP between baseline and 12 month follow-up. No significant independent association with left ventricular systolic function was observed after multivariable adjustment. These associations persisted across sensitivity analyses, the propensity-score-matched cohort, and multiple imputation for missing SPAP data. Effect sizes were modest, and clinical relevance remains uncertain. These hypothesis-generating findings require confirmation in appropriately powered prospective multicenter studies.

## Figures and Tables

**Table 3:** Multivariable linear regression analysis of the association between statin use and echocardiographic changes.

Parameter	Beta	95% CI	*p* Value	Adjusted R^2^
Change in LVEF, %	0.28	(−1.11, 1.66)	0.694	0.523
Change in LVESVi, mL/m^2^	−0.87	(−1.98, 0.24)	0.124	0.503
Change in LVEDVi, mL/m^2^	−0.45	(−2.68, 1.78)	0.689	0.390
Change in mean e’, cm/s	0.75	(0.41, 1.09)	<0.001	0.229
Change in E/A ratio	0.06	(0.01, 0.11)	0.02	0.298
Change in E/e’ ratio	0.19	(−0.18, 0.57)	0.312	0.401
Signed change in SPAP, mmHg	−0.14	(−1.39, 1.11)	0.821	−0.011
Absolute change in SPAP, mmHg	−1.25	(−1.97, −0.53)	<0.001	0.046
Relative change amplitude of estimated SPAP, %	−4.55	(−7.34, −1.76)	0.001	0.131

All models were adjusted for age, sex, body mass index, hypertension, diabetes, dyslipidemia, cumulative doxorubicin-equivalent dose, trastuzumab use, dexrazoxane use, mediastinal radiotherapy, and the baseline value of the corresponding outcome parameter. For SPAP-related models, analyses included 260 patients with complete serial SPAP measurements. *β* coefficients represent the adjusted difference in change between statin users and non-statin users. Abbreviations: CI, confidence interval; LVEF, left ventricular ejection fraction; LVESVi, left ventricular end-systolic volume index; SPAP, systolic pulmonary artery pressure.

**Table 4:** Sensitivity analyses and propensity-score-matched analysis of the association between statin use and echocardiographic changes.

Sensitivity Analysis	LVEF Change *β* (95% CI)	LVEF Change *p*	LVESVi Change β (95% CI)	LVESVi Change *p*	Mean e’ Change *β* (95% CI)	Mean e’ Change *p*	SPAP Relative Change *β* (95% CI)	SPAP Relative Change *p*	Effective *n* (SPAP Analysis)
Primary analysis	0.28 (−1.11, 1.66)	0.694	−0.87 (−1.98, 0.24)	0.124	0.75 (0.41, 1.09)	<0.001	−4.55 (−7.34, −1.76)	0.001	260
Excluding dexrazoxane	−0.18 (−1.77, 1.42)	0.827	−0.57 (−1.82, 0.68)	0.373	0.68 (0.35, 1)	<0.001	−5.22 (−8.44, −2)	0.002	189
Excluding trastuzumab	0.44 (−1.09, 1.96)	0.572	−0.96 (−2.17, 0.24)	0.117	0.83 (0.48, 1.17)	<0.001	−4.52 (−7.54, −1.5)	0.004	215
Excluding mediastinal radiotherapy	−0.24 (−1.79, 1.31)	0.759	−0.59 (−1.8, 0.63)	0.342	0.77 (0.4, 1.15)	<0.001	−5.14 (−8.19, −2.09)	0.001	225
After 1:1 propensity score matching	0.95 (−1.03, 2.93)	0.349	−1.78 (−3.16, −0.4)	0.013	0.84 (0.35, 1.33)	<0.001	−5.67 (−9.37, −1.97)	0.003	144

Values are *β* coefficients with 95% confidence intervals from multivariable linear regression. Primary and exclusion-based analyses were adjusted for demographic, clinical, treatment, and corresponding baseline echocardiographic covariates. The propensity-score-matched analysis included 72 matched pairs (144 patients) and used cluster-robust standard errors at the matched-pair level. Post-matching models additionally adjusted for residual imbalanced covariates. These were age, hypertension, and cardioprotective agent use. The effective *n* column reports patients with complete serial SPAP measurements in each analysis. Abbreviations: CI, confidence interval. LVEF, left ventricular ejection fraction. LVESVi, left ventricular end-systolic volume index. SPAP, systolic pulmonary artery pressure.

**Table 5:** Exploratory subgroup analyses of the association between statin use and change in LVEF.

Subgroup Factor	Subgroup Level	*n*	*β* (95% CI)	*p* Value	*p* for Interaction
Trastuzumab use	No	234	0.44 (−1.09, 1.96)	0.572	0.580
Trastuzumab use	Yes	51	0.01 (−3.53, 3.55)	0.994	0.580
Mediastinal radiotherapy	No	246	−0.24 (−1.79, 1.31)	0.759	0.060
Mediastinal radiotherapy	Yes	39	3.27 (−0.13, 6.67)	0.059	0.060
Cumulative doxorubicin-equivalent dose	<250 mg/m^2^	92	0.34 (−2.21, 2.88)	0.793	0.605
Cumulative doxorubicin-equivalent dose	≥250 mg/m^2^	193	0.42 (−1.31, 2.15)	0.634	0.605

Subgroup analyses were performed by adding an interaction term between statin use and the corresponding subgroup factor to the fully adjusted multivariable model. *p* for interaction refers to the significance of the interaction term. All subgroup analyses were exploratory and not adjusted for multiple testing. Abbreviations: CI, confidence interval; DED, doxorubicin-equivalent dose.

## Data Availability

All data supporting the findings of this study are available within the article and its Supplementary Materials.

## Supplementary Materials

The following supporting information can be downloaded at: https://www.mdpi.com/article/10.3390/jcm15187105/s1, Supplementary Table S1. Standardized mean differences before and after propensity score matching; Supplementary Table S2. Distribution of relative change amplitude of estimated systolic pulmonary artery pressure; Supplementary Table S3. Reproducibility of echocardiographic parameters; Supplementary Table S4. Component analysis of estimated systolic pulmonary artery pressure change; Supplementary Table S5. Comparison of complete-case analysis and multiple imputation for SPAP-related outcomes; Supplementary Figure S1. Love plot of covariate balance before and after propensity score matching; Supplementary Figure S2. Distribution of relative change amplitude of estimated systolic pulmonary artery pressure by statin use.
