# Supplementary material for: Statin Co-Administration Is Associated with Subclinical Echocardiographic Alterations and Relative Change Amplitude of Estimated Systolic Pulmonary Artery Pressure After Anthracycline Chemotherapy: A Retrospective Cohort Study
Source: J Clin Med. 2026 Sep 13;15(18):7105. doi: 10.3390/jcm15187105 (PMC13607195; doi:10.3390/jcm15187105)
Supplement: Supplementary file 1 [file jcm-15-07105-s001.zip › jcm-4512792-supplementary.pdf]

Supplementary Table S1. Standardized mean differences before and after propensity score matching

Covariate	SMD before matching	SMD after matching	Balanced after matching (<0.1)
Age, years	0.276	0.139	No
Male sex	0.388	0.044	Yes
Body mass index, kg/m²	0.193	0.033	Yes
Hypertension	0.016	0.160	No
Diabetes mellitus	0.144	0.049	Yes
Dyslipidemia	0.089	0.041	Yes
Cumulative doxorubicin-equivalent dose, mg/m²	0.061	0.036	Yes
Trastuzumab co-administration	0.082	0.071	Yes
Cardioprotective agent use	0.145	0.161	No
Mediastinal radiotherapy	0.273	0.046	Yes
Baseline LVEF, %	0.452	0.023	Yes
Baseline LVESVi, mL/m²	0.602	0.047	Yes
Baseline LVEDVi, mL/m²	0.409	0.045	Yes
Baseline mean e', cm/s	0.668	0.091	Yes
Baseline estimated SPAP, mmHg	0.173	0.009	Yes

Standardized mean differences (SMDs) were calculated for all 15 covariates included in the propensity score model. An SMD below 0.1 was considered indicative of adequate balance.

Supplementary Table S2. Distribution of relative change amplitude of estimated systolic pulmonary artery pressure

Group	n	Mean ± SD (%)	Median (IQR, %)	Min (%)	Max (%)
Non-statin	110	22.3 ± 13.3	18.4 (12.9, 29)	2.8	59.1
Statin	150	17.3 ± 9.4	15.5 (10.7, 23.8)	0.0	50.0
Total	260	19.4 ± 11.5	16.7 (11.5, 25.8)	0.0	59.1

The distribution of relative change amplitude of estimated systolic pulmonary artery pressure is summarized for statin users and non-statin users. Analyses included 260 patients with complete serial SPAP measurements. Abbreviations: IQR, interquartile range; SD, standard deviation; SPAP, systolic

pulmonary artery pressure.

Supplementary Table S3. Reproducibility of echocardiographic parameters

Echocardiographic parameter	Intra-observer ICC (95% CI)	Inter-observer ICC (95% CI)	Bland-Altman mean difference (95% limits of agreement)
Left ventricular systolic parameters			
LVEF, %	0.94 (0.88, 0.97)	0.92 (0.84, 0.96)	0.4% (-2.1%, 2.9%)
LVEDVi, mL/m²	0.93 (0.86, 0.97)	0.91 (0.82, 0.96)	1.1 mL/m² (-2.8, 5.0 mL/m²)
LVESVi, mL/m²	0.92 (0.85, 0.96)	0.90 (0.81, 0.95)	0.8 mL/m² (-1.9, 3.5 mL/m²)
Diastolic parameters			
E wave, cm/s	0.91 (0.83, 0.96)	0.89 (0.79, 0.94)	1.2 cm/s (-4.5, 6.9 cm/s)
E/A ratio	0.87 (0.75, 0.93)	0.84 (0.70, 0.92)	0.03 (-0.18, 0.24)
mean e', cm/s	0.90 (0.81, 0.95)	0.88 (0.78, 0.94)	0.2 cm/s (-0.8, 1.2 cm/s)
Pulmonary hemodynamic parameters			
TRPG, mmHg	0.89 (0.79, 0.95)	0.87 (0.75, 0.93)	0.2 mmHg (-1.8, 2.2 mmHg)
RAP, mmHg	0.85 (0.72, 0.92)	0.83 (0.69, 0.91)	0.1 mmHg (-1.5, 1.7 mmHg)
SPAP, mmHg	0.88 (0.77, 0.94)	0.86 (0.74, 0.93)	0.3 mmHg (-2.07, 2.67 mmHg)

Thirty randomly selected studies were remeasured by two independent blinded sonographers. Intra-observer and inter-observer intraclass correlation coefficients (ICCs) with 95% confidence intervals are reported. Bland-Altman analysis was used to calculate mean differences and 95% limits of agreement. Abbreviations: LVEF, left ventricular ejection fraction; LVEDVi, left ventricular end-diastolic volume index; LVESVi, left ventricular end-systolic volume index; TRPG, tricuspid regurgitant pressure gradient; RAP, right atrial pressure; SPAP, systolic pulmonary artery pressure.

Supplementary Table S4. Component analysis of estimated systolic pulmonary artery pressure change

Parameter	Non-statin baseline	Non-statin 12-month	Non-statin change	Statin baseline	Statin 12-month	Statin change	P value (change)
Tricuspid regurgitant pressure gradient (TRPG), mmHg	22.5 ± 3.8	25.9 ± 6.7	3.3 ± 5.8	22 ± 3.9	25.2 ± 5.3	3.2 ± 4	0.820
Right atrial pressure (RAP), mmHg	4.2 ± 1.9	4.4 ± 1.9	0.2 ± 0.6	5.3 ± 2.2	5.3 ± 2.1	0.1 ± 0.4	0.026
Systolic pulmonary artery pressure (SPAP), mmHg	26.7 ± 3.2	30.3 ± 6.1	3.6 ± 5.7	27.3 ± 3.3	30.6 ± 4.9	3.3 ± 4	0.634

Changes in tricuspid regurgitant pressure gradient and estimated right atrial pressure were compared between statin users and non-statin users. P values refer to between-group comparisons of change from baseline using independent samples t-tests. Abbreviations: TRPG, tricuspid regurgitant pressure gradient; RAP, right atrial pressure; SPAP, systolic pulmonary artery pressure.

Supplementary Table S5. Comparison of complete-case analysis and multiple imputation for SPAP related outcomes

Analysis	Outcome	β (95% CI)	P_value	N
Complete-case analysis (n=260)	Signed change in SPAP, mmHg	-0.14 (-1.39, 1.11)	0.821	260
Complete-case analysis (n=260)	Absolute change in SPAP, mmHg	-1.25 (-1.97, -0.53)	<0.001	260
Complete-case analysis (n=260)	Relative percentage change in SPAP, %	-4.55 (-7.34, -1.76)	0.001	260
Multiple imputation (MICE, n=285)	Signed change in SPAP, mmHg	-0.14 (-1.36, 1.08)	0.819	285
Multiple imputation (MICE, n=285)	Absolute change in SPAP, mmHg	-1.22 (-1.94, -0.5)	0.001	285
Multiple imputation (MICE, n=285)	Relative percentage change in SPAP, %	-4.49 (-7.27, -1.71)	0.002	285

Multiple imputation by chained equations was performed using all baseline demographic, clinical, treatment, and echocardiographic covariates, including all 285 patients. Results from the imputed full cohort were compared with the primary complete-case analysis including 260 patients. Abbreviations: CI, confidence interval; SPAP, systolic pulmonary artery pressure; MICE, multiple imputation by chained equations.

Supplementary Figure S1. Love plot of covariate balance before and after propensity score matching

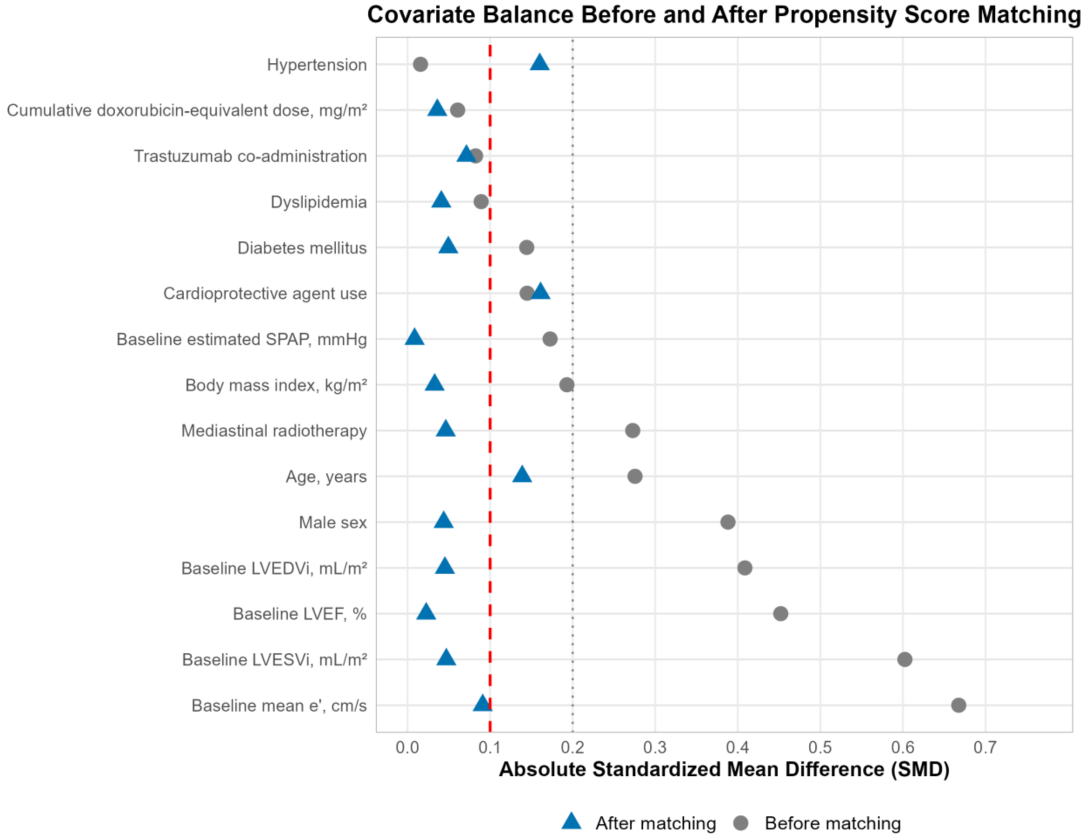

Gray circles represent absolute standardized mean differences before matching, and blue triangles represent absolute standardized mean differences after 1:1 nearest-neighbor propensity score matching. The vertical dashed line indicates the SMD = 0.1 threshold for adequate balance. All baseline echocardiographic parameters included in the matching model are shown.

Supplementary Figure S2. Distribution of relative change amplitude of estimated systolic pulmonary artery pressure by statin use

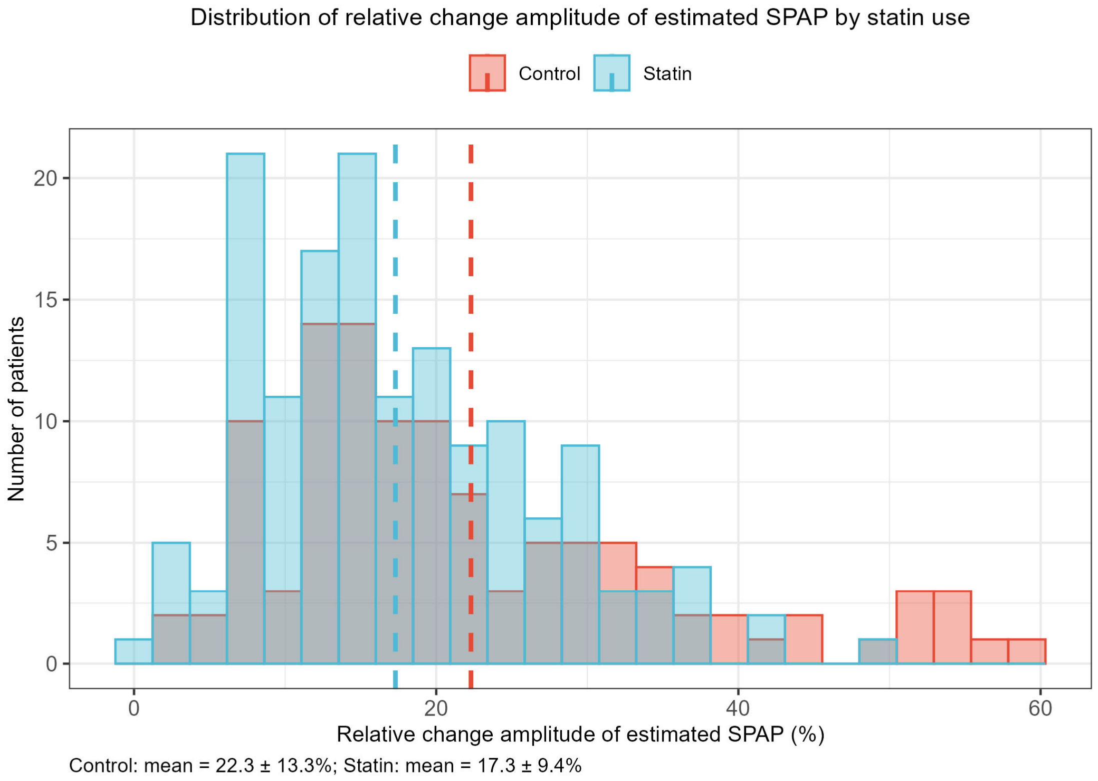

Histogram showing the distribution of relative change amplitude of estimated systolic pulmonary artery pressure in non-statin users (red) and statin users (blue). The unadjusted mean relative change amplitude was 22.3 ± 13.3% in non-statin users and 17.3 ± 9.4% in statin users. Analyses included 260 patients with complete serial SPAP measurements.
